# The domestication of the African Union model law on medical products regulation: Perceived benefits, enabling factors, and challenges

**DOI:** 10.3389/fmed.2023.1117439

**Published:** 2023-01-30

**Authors:** Bakani Mark Ncube, Admire Dube, Kim Ward

**Affiliations:** School of Pharmacy, University of the Western Cape, Bellville, South Africa

**Keywords:** pharmaceutical policy, consolidated framework for implementation research (CFIR) of Damschroder et al., medicines regulatory harmonisation, African medicines regulatory harmonisation initiative, AU Model Law on Medical Products Regulation, African Medicines Agency

## Abstract

**Introduction:**

In 2016, the African Union (AU) Model Law on Medical Products Regulation was endorsed by AU Heads of State and Government. The aims of the legislation include harmonisation of regulatory systems, increasing collaboration across countries, and providing a conducive regulatory environment for medical product/health technology development and scale-up. A target was set to have at least 25 African countries domesticating the model law by 2020. However, this target has not yet been met. This research aimed to apply the Consolidated Framework for Implementation Research (CFIR) in analysing the rationale, perceived benefits, enabling factors, and challenges of AU Model Law domestication and implementation by AU Member States.

**Methods:**

This study was a qualitative, cross-sectional, census survey of the national medicines regulatory authorities (NRAs) of Anglophone and Francophone AU Member States. The heads of NRAs and a senior competent person were contacted to complete self-administered questionnaires.

**Results:**

The perceived benefits of model law implementation include enabling the establishment of an NRA, improving NRA governance and decision-making autonomy, strengthening the institutional framework, having streamlined activities which attract support from donors, as well as enabling harmonisation, reliance, and mutual recognition mechanisms. The factors enabling domestication and implementation are the presence of political will, leadership, and advocates, facilitators, or champions for the cause. Additionally, participation in regulatory harmonisation initiatives and the desire to have legal provisions at the national level that allow for regional harmonisation and international collaboration are enabling factors. The challenges encountered in the process of domesticating and implementing the model law are the lack of human and financial resources, competing priorities at the national level, overlapping roles of government institutions, and the process of amending/repealing laws being slow and lengthy.

**Conclusion:**

This study has enabled an improved understanding of the AU Model Law process, the perceived benefits of its domestication, and the enabling factors for its adoption from the perspective of African NRAs. NRAs have also highlighted the challenges encountered in the process. Addressing these challenges will result in a harmonised legal environment for medicines regulation in Africa and be an important enabler for the effective operation of the African Medicines Agency.

## 1. Introduction

The African continent has 55 countries and is home to a collective 1.2 billion people (1). All countries in Africa, except Sahrawi Republic, have a national medicines regulatory authority (NRA) or an administrative unit conducting some or all functions expected of an NRA (1). One country, the United Republic of Tanzania, has two NRAs–the Tanzania Medicines and Medical Devices Authority (TMDA) and the Zanzibar Food and Drug Agency (ZFDA) (2). African NRAs have different organisational set-ups and functionalities (1, 2). Some operate as departments or units under their respective Ministry responsible for Health, whereas others are semi-autonomous (1, 2). Only 7% of the NRAs on the continent have moderately developed capacity and over 90% have minimal-to-no-capacity (2). Medicine regulation creates a critical link between access and quality (3). It is therefore important that countries have well-functioning regulatory systems as they underpin the safety, quality and efficacy of medical products/health technologies (3). Regulatory systems also prevent the circulation of substandard and falsified medical products on the market and facilitate cost-effective and rational medicine use (2, 3).

The foundation for regulation is medicines laws (2). The legislation for medical products must be comprehensive as well as cover all pharmaceutical sector activities and a wide range of medical products (2). They must also provide the NRA with adequate powers to control and regulate the pharmaceutical market. In sub-Saharan Africa, 40 out of 46 countries have legislation for medicines and only 15% of the NRAs have a legal mandate to perform all critical regulatory functions (2). The critical functions include marketing authorisation, licencing of pharmaceutical manufacturers, import and export control, market surveillance, quality control, and clinical trials oversight. The legislation in African countries has commonalities and disparities and varies in capacity and implementation. Some of the differences are in the degree of comprehensiveness, scope of products being regulated, and NRA functions and practices (2). Therefore, convergence towards a common medicines regulatory framework is needed which will also facilitate benchmarking among African countries.

**FIGURE 1:**
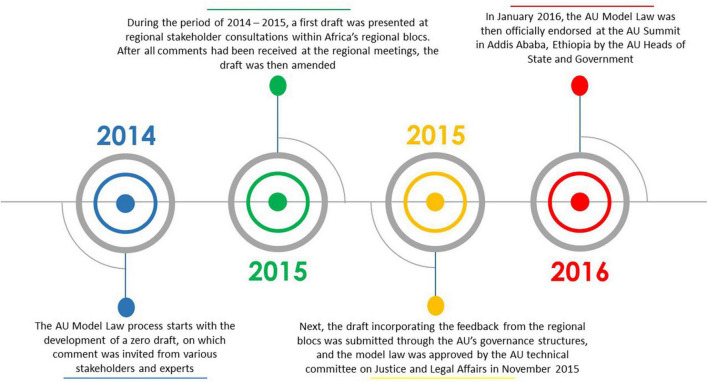
Timeline of the AU Model Law development process.

Over the years, the African Union (AU) has provided support for medicines regulatory harmonisation and some regional economic communities (RECs) in Africa at present have streamlined regulatory systems (4). Fully exploiting this momentum, the African Union Development Agency–New Partnership for Africa’s Development (AUDA-NEPAD) and key stakeholders developed the AU Model Law on Medical Products Regulation in 2014, hereafter referred to as the AU Model Law. The model law is a non-prescriptive legislation meant to be domesticated and implemented by AU Member States and RECs to harmonise regulatory systems, increase collaboration across countries, and provide a regulatory environment that is conducive for health technology development and scale-up (4–8). It provides a template for African countries to harmonise their regulatory frameworks and it outlines the key functions and standards that should form part of the regulatory system (9). Access to quality-assured, safe and efficacious medical products/health technologies has been a significant challenge in Africa for decades, partly due to weak or absent regulatory systems, and the intention with the AU Model Law is to also catalyse access to these lifesaving medical products (4, 5, 7). In addition, the AU Model Law is meant to support countries to incorporate powers to levy, collect, and use fees for services rendered when reviewing or enacting their laws. Furthermore, the model law is meant to deliver an enabling regulatory environment for the pharmaceutical industry to provide the African population with medical products (10, 11). This supports the AU’s desire to promote local pharmaceutical production (6). Figure 1 shows a timeline of the AU Model Law development process. This process, according to a United Nations Development Programme (UNDP) publication, is not a standalone development; it complements partnerships, regional integration ventures, the incorporation of global best practices in medical products regulation, the Pharmaceutical Manufacturing Plan for Africa (PMPA) as well as the Roadmap for Shared Responsibility for the AIDS, Tuberculosis and Malaria Response in Africa (5). All these elements will potentially ensure the relevance and sustainability of the AU Model Law (5).

The AU Model Law, which is available in English, French, Portuguese, and Arabic, consists of a Preamble and ten Parts (9). The Parts are then divided into 35 Articles (9). Through the process of AU Model Law domestication, African countries can either adopt the model law as is or adapt it so that it is consistent with their constitutional principles and legal system, as well as amend or repeal any inconsistent national laws (4, 7, 12, 13). “Domestication” is defined as “the legislative action taken to incorporate, into the national legislation, an agreement or treaty of a regional, continental, or international institution” (9). Although the model law is not a treaty, it requires a similar act of domestication for it to become domestically binding in the AU Member State (9). Therefore, a process is needed at the national level in the individual AU Member States to align the national law with the AU Model Law (9). The AU Model Law is considered to have been domesticated if the country’s regulatory law is already in alignment with the model law, or if the model law is adopted by the country verbatim as its regulatory law, or if all the essential provisions of the AU Model Law are adopted by the country (9).

The AUDA-NEPAD wrote the “African Medicines Regulatory Harmonisation (AMRH) Strategic Framework (2016–2020)” (14) which builds on previous harmonisation efforts and was meant to offer ongoing support to AUDA-NEPAD and its partners (14, 15). Strategic Direction I of this document is on policy alignment and regulatory reforms, and some of the targets related to it include having at least three regions adopting regional policies and legal frameworks for the regulation of medical products by 2020 (16), and at least 25 countries domesticating the AU Model Law by 2020 (5, 13, 16). To accelerate the achievement of these targets, the AMRH initiative established the Technical Working Group on Medicines Policy and Regulatory Reforms (TWG-MPRR) to support and guide the domestication process (9). The AUDA-NEPAD has also been coordinating legal capacity building and providing technical support to enable African countries to review their existing legislation on medical products regulation and make the required amendments for them to align with the AU Model Law (9). Despite these efforts, the implementation targets for the AU Model Law were not met. Several African countries did, however, manage to adopt or adapt the AU Model Law by the target date and they could potentially offer lessons and best practices that can be emulated when revising national medicines regulatory systems using the AU Model Law as the reference document. These countries also offer examples of domesticating and implementing a version of the AU Model Law that best responds to a country’s respective needs in order to set up a streamlined regulatory system that ensures that medical products meet international standards of quality, safety and efficacy. There is a need to understand the current status of AU Model Law domestication and implementation in order to provide a foundation for identifying the existing gaps and opportunities for improving the regulation of medical products in Africa, public health protection and promotion, and pharmaceutical industry advancement on the continent. Therefore, the aim of this research was to apply the Consolidated Framework for Implementation Research (CFIR) in analysing the rationale, perceived benefits, enabling factors, and challenges of AU Model Law domestication and implementation by AU Member States.

## 2. Study objectives

The study objectives were:

1. To determine the perceived benefits of domesticating and implementing the AU Model Law by AU Member States.
2. To determine the challenges encountered by AU Member States in domesticating and implementing the AU Model Law.
3. To assess the enabling factors for AU Model Law domestication and implementation in AU Member States that have done so.

## 3. Materials and methods

This study was a qualitative, cross-sectional, census survey of the NRAs of Anglophone and Francophone AU Member States. NRAs that do not participate in the AMRH initiative were excluded from the study as the contact details of the Head of NRA or an AMRH initiative liaison were not available in the initiative’s database. These countries are Djibouti, Libya, Malawi, Mauritius, and Sahrawi Republic. Rwanda was also excluded from the main survey as the research instruments were piloted on the Rwanda Food and Drugs Authority.

The NRAs of the remaining 45 African jurisdictions were included in the study and two regulatory officials, viz., the Head of the NRA and their Chief Regulatory Officer (or an alternative senior competent person) from each NRA were purposively sampled and contacted *via* electronic mail to complete the questionnaire on Survey Monkey. The questionnaire developed for this study consisted of closed- and open-ended questions that elicited perceptions on the rationale and motivation for AU Model Law domestication and implementation and the factors that enable and pose challenges to domestication and implementation of the law. Depending on the official language spoken in the recipient’s country, self-administered questionnaires, the accompanying information and consent documents were provided in either English or French. Participants were given 6 weeks (between October and November 2021) to complete and submit the questionnaires and four reminder emails with the Survey Monkey link were sent out during this period. As the survey involved high-level participants, AMRH initiative staff at the AUDA-NEPAD were engaged to support this research and facilitate access to NRAs in the data collection phase. The study was approved by the HSSREC, University of the Western Cape, South Africa (HSSREC Reference Number: HS21/5/39).

### 3.1. Data analysis

**FIGURE 2:**
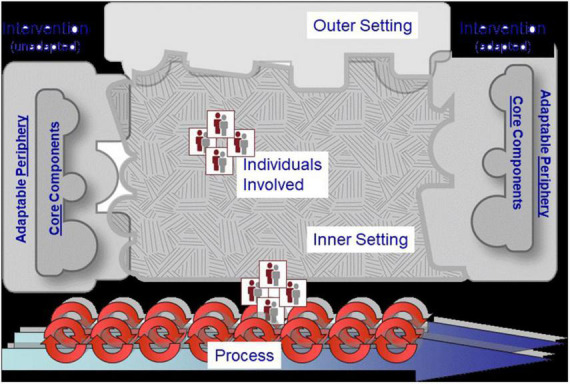
The Consolidated Framework for Implementation Research (CFIR) of Damschroder et al. (17).

The qualitative data were subjected to *a priori* coding and thematic analysis. Deductive analysis was done using the Consolidated Framework for Implementation Research (CFIR) of Damschroder et al. (17) which served as the conceptual and analytical framework for gaining a comprehensive understanding of the implementation of the AU Model Law in AU Member States. The CFIR is a meta-theoretical framework that provides a menu of constructs associated with effective implementation and includes taxonomy, terminology, and definitions that create a knowledge base of implementation factors across multiple contexts (18). The CFIR identifies constructs across five domains that should be considered when implementing an intervention: (i) the characteristics of the intervention that is implemented, (ii) the outer setting or factors such as the economic, political, and social context within which an organisation resides, (iii) the inner setting, or features such as the structural, political, and cultural contexts through which the implementation process will proceed, (iv) characteristics of individuals involved with the intervention and/or implementation process; and (v) process which is related to essential activities of the implementation process that are common across organisational change models. This framework lends itself well to the domestication and implementation of the AU Model Law because it provides a practical approach for the systematic assessment of perceived benefits, processes, facilitators, and potential barriers encountered in the implementation of an innovation. It can also be easily adapted to suit diverse settings and scenarios, including low-income contexts. Figure 2 illustrates the CFIR and its main domains.

## 4. Results

### 4.1. Overview of legislation for medicines regulation in Africa

Twenty-six completed questionnaires were received from 21 NRAs. 69% (*n* = 18) of these questionnaires were from NRAs in Anglophone countries [Botswana, Ethiopia, Ghana, Kenya, the Kingdom of Eswatini, Liberia, Namibia, Seychelles, Sierra Leone, South Sudan, Tanzania (mainland), Tanzania (Zanzibar), The Gambia, and Zimbabwe] and the remaining 31% (*n* = 8) of the questionnaires were from NRAs in Francophone countries (Burundi, Cape Verde, Comoros Islands, Ivory Coast, Niger, Togo, and Tunisia). No responses were received from Algeria, Benin, Burkina Faso, Cameroon, Central African Republic, Chad, Congo Republic, Democratic Republic of Congo, Egypt, Equatorial Guinea, Eritrea, Gabon, Guinea, Lesotho, Madagascar, Mali, Mauritania, Morocco, Nigeria, Senegal, Somalia, South Africa, Sudan, Uganda, and Zambia. This study therefore had 47% of the NRAs participating in the research and a 29% response rate from the participating officials.

All the countries in this study had an NRA or an administrative unit that is responsible for the regulation of medical products. 95% of the NRAs that participated in this survey (*n* = 20) stated that there is legislation in place for medicines regulation. One country (Seychelles) does not have legislation for medicines regulation. In some countries, legislation for medicines regulation dates back as far as 1957 whereas in other countries, legislation first came into effect as recently as 2020. Most countries have updated their legislation at least once and some are currently doing so. Table 1 provides an overview of study countries that have domesticated the AU Model Law and Table 2 provides an overview of study countries that have not yet domesticated the model law.

**TABLE 1:** Study countries that have domesticated the AU Model Law (*n* = 6).

AU member state	NMRA	Title of legislation	National legislation introduced/Updated^a^
Burundi	Autorité Burundaise de Régulation des Médicaments à usage humain et des Aliments (ABREMA)	Loi N°1/11 du 8 Mai 2020 portant réglementation de l’exercice de la pharmacie et du médicament à usage humain	2020
Cote d’Ivoire	Autorité Ivoirienne de Régulation Pharmaceutique (AIRP)	Loi 2017-541 du 03 Aout 2017	-/2017
Kenya	Pharmacy and Poisons Board	The Pharmacy and Poisons Board Act, CAP 244	1957/2019
Tanzania (mainland)	Tanzania Medicines and Medical Devices Authority	Tanzania Medicines and Medical Devices Act, CAP 219	2003/2019
Tanzania (Zanzibar)	Zanzibar Food and Drug Agency (ZFDA)	Zanzibar Food, Drug And Cosmetics Act #2/06 and its Amendment #3/17	2007/2017
The Gambia	Medicines Control Agency	Medicines and Related Products Regulations 2020	2015/2020
Tunisia	Direction de la Pharmacie et du Médicament	Loi 85–91 réglementant la fabrication et l’enregistrement des médicaments humains Loi 78–23 relative à la pharmacie vétérinaire	1969/2020

ABREMA, Autorité Burundaise de Régulation des Médicaments à usage humain et des Aliments; AIRP, Autorité Ivoirienne de Régulation Pharmaceutique; ZFDA, Zanzibar Food and Drug Agency (ZFDA).

^a^This is the year when the country introduced its legislation for medicines regulation or updated the existing legislation and not the year when the AU Model Law was domesticated or implemented.

**TABLE 2:** Study countries that have not domesticated the AU Model Law (*n* = 15).

AU member state	NMRA	Title of legislation	National legislation introduced/Updated^a^
Botswana	Botswana Medicines Regulatory Authority (BoMRA)	Medicines and Related Substance Act of 2013	2013/Amendment of the Act is ongoing
Cape Verde	Entidade Reguladora Independente da Saúde (ERIS)	Decreto - lei n° 59/2006 de 26 de décembre, que réglemente l’autorisation de mise sur le marché, l’enregistrement, la fabrication, l’importation, la commercialisation et le publicité de médicaments à usage humain	1993/2006
Comoros^b^	Agence Nationale des Médicaments et des Evacuations Sanitaires (ANAMEV)	Code de la Santé Publique, Livre V	1995/2020
Ethiopia	Ethiopian Food and Drug Authority	Food and Medicine Regulation, Proclamation 1112/2019	1999/2019
Ghana^c^	Food and Drugs Authority	Public Health Act, 2012 (ACT 851)–Part 7	1992/2012
Kingdom of Eswatini	Ministry of Health–Medicines Regulatory Unit (MoH-MRU)	Medicines and Related Substances Control Act No. 9 of 2016	2016/2020
Liberia	Liberia Medicines and Health Products Regulatory Authority	An Act to Establish the Liberia Medicines and Health Products Regulatory Authority (LMHRA) of 2010	2010
Namibia	Namibia Medicines Regulatory Council	Medicines and Related Substances Control Act, Act 13 of 2003	2003/2007
Niger^d^	Direction de la Pharmacie et de la Médecine Traditionnelle	Loi N°97-05 du 02 Juin 1997 Portant Ratification de l’Ordonnance 97-05 Portant Législation Pharmaceutique	1997/2021
Seychelles	Medicine Regulatory Unit, Public Health Authority	Not applicable	Not applicable
Sierra Leone	Pharmacy Board of Sierra Leone	Pharmacy and Drugs Act 2001	1988/2001^e^
South Sudan	South Sudan Drug and Food Control Authority	South Sudan Drug and Food Control Authority Act 2012	2012
Togo	Direction de la Pharmacie, du Médicament et des Laboratoires (DPML)	Loi n° 2009-007 du 15 mai 2009 portant code de la Santé Publique de la République togolaise. (Titre IV: du médicament, des dispositifs médicaux et de la pharmacie)	2009
Zimbabwe	Medicines Control Authority of Zimbabwe	Medicines and Allied Substances Control Act 15:03	1969/1997

ANAMEV, Agence Nationale des Médicaments et des Evacuations Sanitaires; BoMRA, Botswana Medicines Regulatory Authority; DPML, Direction de la Pharmacie, du Médicament et des Laboratoires; ERIS, Entidade Reguladora Independente da Saúde; LMHRA, Liberia Medicines and Health Products Regulatory Authority; MoH-MRU, Ministry of Health–Medicines Regulatory Unit.

^a^This is the year when the country introduced its legislation for medicines regulation or updated the existing legislation and not the year when the AU Model Law was domesticated or implemented.

^b^Process currently at the level of the Ministry of Health.

^c^Ghana is currently in the process of domesticating the model law.

^d^The revision of the 1997 law is in progress. This revision considers the domestication of the model law.

^e^It has been updated but not yet approved in 2021 to address current emerging issues in tandem with the AU model law.

This study found that countries update their legislation for medicines regulation for reasons such as the desire to establish a new regulatory authority, to transform the existing regulatory authority, or to align their legislation with the AU Model Law and international best practices. 33% (*n* = 7) of NRAs reported that they have domesticated the AU Model Law and 93% (*n* = 13) of the countries that have not domesticated the model law stated that despite having not domesticated the model law, they have an intention to do so. Only one NRA indicated that they have no interest in domesticating the model law because their law, which came into effect a few years before the model law was developed, already had all the components of the AU Model Law.

The results are presented according to the domains of the CFIR. Four of the five domains are consistent with our study findings and these domains are intervention characteristics, outer setting, inner setting, and process. Only the constructs and sub-constructs of these four domains that are consistent with our study findings will be presented. None of the participants’ responses aligned with any of the constructs in the “characteristics of individuals” domain. Sample participant quotes are provided to support the findings.

### 4.2. Intervention characteristics

The study findings are consistent with three out of eight intervention characteristic constructs.

#### 4.2.1. Evidence strength and quality

This construct deals with stakeholders’ perceptions of the quality and validity of evidence supporting the belief that the intervention will have desired outcomes.

In this research study, 35% (*n* = 9) of the respondents considered the harmonisation of regulatory systems and enabling cooperation with other NRAs to be a benefit of domesticating and implementing the model law. One respondent stated that “*aligning with the AU Model Law will make regional and continental harmonisation easier. Since the AU Model Law is comprehensive, it ensures that all aspects of medicines regulation and control are covered. It may also facilitate mutual recognition between and amongst countries*” (P5). Other participants shared similar sentiments as they stated that the model law was expected to “*fill the gaps in the current Act as well as to allow regional harmonisation*” (P17), “*support harmonisation of the data requirements for evidence of quality, safety, and efficacy of medical products across the sub-region*” (P8), and to bring about a “*wider scope of regulated products, and alignment to regional and international laws that would enable harmonisation initiatives*” (P10).

Other common perceived benefits of domesticating the model law include being “*in line with regional international standards and best practices*” (P4), “*facilitating the exchange of regulatory information*” (P6), “*an increased number of registered medical products*” (P6), “*improving the regulation of medical products and technologies*” (P8), curbing the circulation of substandard and falsified medical products, and having an NRA that is “*fully mandated to conduct regulatory activities*” (P15). One participant (P15) felt that domesticating and implementing the model law would also enable the regulated community to clearly understand their roles.

In addition, the model law’s domestication and implementation was perceived by respondents to result in a strong, autonomous regulatory authority (P22), “*improve transparency and efficiency of the medicines regulatory framework and safety monitoring systems*” (P8) and enable countries to have appropriate laws that include all regulatory functions expected of an NRA (P21). This ensures that medicines distributed in countries are safe, efficacious and of good quality. For countries with limited resources, it was expressed that the “*AU Model Law was timely as it enabled (them) to adopt strong pharmaceutical laws in a rapid manner*” (P21).

Furthermore, one participant perceived the model law “*to outline and put regulations in proper perspectives*” (P2), i.e., it would expand policies, result in a coordinated approach for medicines regulation, enable the evaluation of incoherent policy frameworks, and enable efficient and aligned frameworks to be developed. A participant from a different country considered domestication and implementation to result in “*better oversight of clinical trials, increased export opportunities for domestic pharmaceutical manufacturers, increased confidence in the health system and medicines, and reduced antimicrobial resistance*” (P11).

Moreover, one participant voiced that for them, being the first country in the region to domesticate the AU Model Law was considered beneficial as it would bring attention to their NRA and enable them to participate in regional and continental harmonisation initiatives (P7). Another participant thought that AU Model Law domestication would allow them to participate in the realisation of the African Medicines Agency project (P22).

Stakeholders’ perceptions of the quality and validity of evidence supporting the belief that the intervention will have desired outcomes are corroborated by seven NRAs in six African countries that have implemented the AU Model Law who report that they are accruing benefits from implementation. Table 3 highlights participants’ perceived benefits of implementing the AU Model Law. These include enabling the establishment of an NRA, improving NRA governance and decision-making autonomy, strengthening the institutional framework, having streamlined activities which attract support from donors, as well as enabling harmonisation, reliance, and mutual recognition mechanisms. All participants who stated that they have implemented the AU Model Law reported that there have been no disadvantages to its implementation.

**TABLE 3:** The benefits of AU Model Law implementation reported by seven African national medicines regulatory authorities (NRAs) (*N* = 9).

Participant	AU member state	Benefits accrued from AU Model Law implementation
P22	Tunisia	The participant had no benefits to report at this stage.
P23	Cote d’Ivoire	• Better governance • Management autonomy, decision-making autonomy • Strengthening of the institutional framework
P21	Burundi	• Creation of ABREMA with clear missions for each service allowing the smooth running of regulatory functions • With the pricing of services, not yet in place, ABREMA will have financial resources allowing it to implement its mission • The pharmaceutical sector is well regulated • The reduction of dependence on technical and financial partners in the regulations
P10	Kenya	• Increased revenue streams • It has enabled harmonisation, reliance, and mutual recognition mechanisms
P12	Kenya	• Cooperation with other regional, continental, and international institutions therefore saving time taken to make regulatory decisions • Provided a framework for improving regulation of medicines • Transparency and accountability increased as the functions and powers of the NRA are clearly stipulated in law • Effective governance of the NRA as the CEO is appointed by the Board
P26	Kingdom of Eswatini	• The provisions on making use of regulatory decisions made in other jurisdictions have been of particular benefit to Eswatini as a country with limited regulatory capacity
P9	Tanzania (mainland)	• Alignment of the regulatory activities with other agencies and international organisations such as WHO • Having streamlined activities which attract support from donors • It has led to adequate systems for ensuring the quality, safety, and efficacy of medicines, medical devices, and other health technologies
P7	Tanzania (Zanzibar)	• Harmonisation initiatives in EAC, twinning programmes, joint regulatory activities • The AU Model Law strengthened ZFDA’s regulatory functions
P4	The Gambia	• Establishment and capacity building of the NRA to perform regulatory activities

ABREMA, Autorité Burundaise de Régulation des Médicaments à usage humain et des Aliments; AU, African Union; CEO, Chief Executive Officer; EAC, East African Community; NRA, national medicines regulatory authority; WHO, World Health Organization; ZFDA, Zanzibar Food and Drug Agency.

#### 4.2.2. Adaptability

**FIGURE 3:**
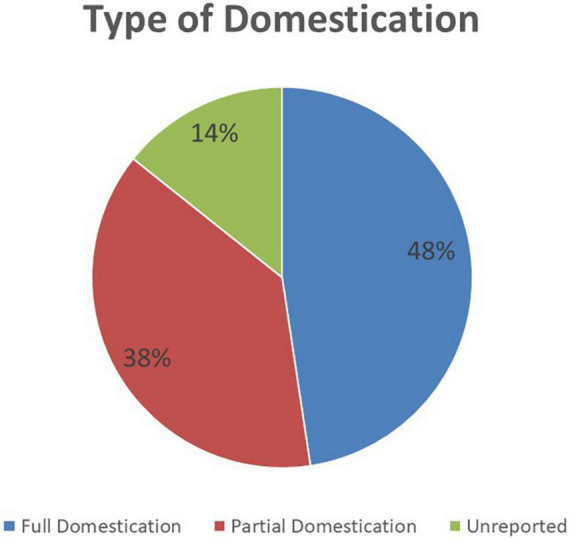
The type of AU Model Law domestication performed or being performed by 21 African national medicines regulatory authorities (NRAs) (*N* = 21).

**FIGURE 4:**
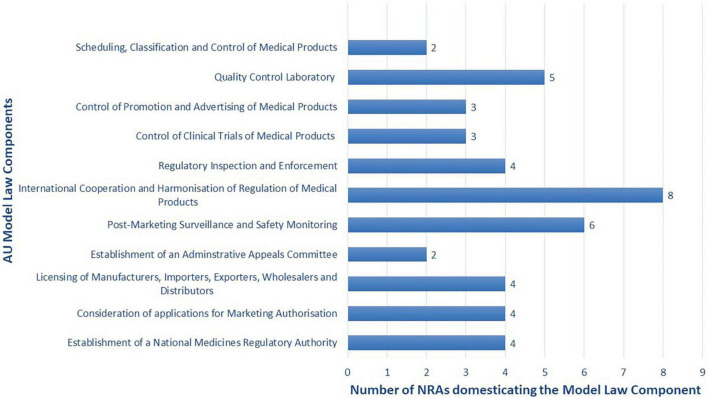
Components of the model law adopted or being adopted by the eight African national medicines regulatory authorities (NRAs) performing a partial domestication (*N* = 8).

Adaptability is the degree to which an intervention can be adapted, tailored, refined, or reinvented to meet local needs. The AU Model Law is adaptable as countries can either domesticate it partially or in full to meet their needs. 48% (*n* = 10) of the NRAs are reported to have domesticated or to be domesticating the AU Model Law in full. These are NRAs of Botswana, Burundi, Cote d’Ivoire, the Kingdom of Eswatini, Liberia, Sierra Leone, Tanzania (mainland), The Gambia, Tunisia, and Zimbabwe. A total of 38% (*n* = 8) of the NRAs are reported to have domesticated or to be domesticating the AU Model Law partially, and these are national regulators of Comoros Islands, Ghana, Kenya, Namibia, Niger, Seychelles, Tanzania (Zanzibar), and Togo. All of these countries’ regulatory authorities are adopting the component that allows for international cooperation and harmonisation of regulation of medical products. The components least adopted are for the establishment of an administrative appeals committee and for scheduling, classification, and control of medical products. The remaining 14% (*n* = 3) of NRAs are uncertain about which type of domestication they will conduct. Figure 3 shows the type of AU Model Law domestication performed or being performed by NRAs in Africa and Figure 4 illustrates the components of the model law adopted by NRAs performing a partial domestication. According to participants, full domestication of the AU Model Law was or is being done for the reasons outlined below.

*“Full domestication was chosen to close some of the gaps identified by the WHO Global Benchmarking Tool (GBT) assessment and to clarify other provisions that were in the current Act after benchmarking with the AU Model Law”* (P15).

*“To harmonise the regulatory procedures of (our country) with those of the Member States of the African Union”* (P21).

*“This law meets our expectations in terms of pharmaceutical regulation”* (P23).

*“The AU Model Law was found to contain all the provisions that were seen as necessary for the regulation of medicinal products in the country. It was also thought that aligning the country’s legislation to the AU Model Law would make it easier to participate in regional harmonisation initiatives on the regulation of medicinal products”* (P26).

*“As it relates to medicines regulations; to provide a framework to guide, strengthen the regulatory environment for the delivery of quality, safe, and efficacious medicines. To accelerate access to lifesaving interventions to improve health impact”* (P2).

*“To follow international best practice”* (P11).

Reasons for partial domestication of the AU Model Law mentioned by the participants are outlined below.

*“There was a need for enhanced regulatory harmonisation”* (P18).

*“(Our country) is implementing a partial domestication because most of the provisions in the model law are already covered in the Public Health Act”* (P8).

*“The rest of the regulatory functions existed before the domestication of the AU Model Law. The (NRA) is an existing regulatory authority. Amendments served to align (the medicines legislation) to the AU Model Law and to widen the scope”* (P10).

*“The intention is to enact a more comprehensive legislation to deal with the regulation of health products and technologies”* (P12).

*“Currently, there are a lot of omissions and loopholes in the Act, and they can be addressed by the sections in the AU Model Law”* (P17).

*“We have a law in place; the partial implementation is to include the provisions that are missing and to make some more comprehensive”* (P5).

*“The domestication of the model law will make it possible to put in place an adequate framework for the circulation of medical products of safe and effective quality”* (P20).

*“(Our country) is too small to establish a National Medicine Regulatory Authority. Instead, a Medicine Regulatory Service will be established as a section under the Public Health Authority”* (P13).

#### 4.2.3. Complexity

Complexity refers to the perceived difficulty of implementation, reflected by duration, scope, radicalness, disruptiveness, centrality, and intricacy and number of steps required to implement. Most respondents stated that there were no perceived disadvantages of domesticating the model law. However, those that did considered the lengthy process of amending existing Acts to be a disadvantage. Participants also stated that model law domestication is a *“cumbersome process as change of legislation is onerous”* (P16) and *“amending laws is a slow process especially if there are no identified persons/institutions to push the agenda forward internally”* (P26).

In addition, one participant was concerned that *“since the provisions of the AU Model Law will be applied across the region, there is the possibility of (their country) relying on data from other member states by reason of harmonisation”* (P8) and the *“data might not be up to scratch”* (P8). Other perceived disadvantages that were reported include the fact that *“the expanded mandate (brought about by the AU Model Law) may not be affordable”* (P5) and *“regulated products are not common across the region, specifically the regulations for food”* (P10). It was also stated that the AU Model Law *“does not address the issue of the management of unusable (expired) pharmaceutical products”* (P23) and it also *“does not address the question of other regulated products, in particular cosmetic products, dietetic products, food supplements, etc.”* (P23).

Furthermore, the fact that *“the country should retain sovereignty in deciding what to regulate”* (P10) was considered a disadvantage of domesticating and implementing the model law.

### 4.3. Outer setting

The study findings are consistent with two out of four outer setting constructs.

#### 4.3.1. Cosmopolitanism

Cosmopolitanism is the degree to which an organisation is networked with other external organisations. In this research study, it was reported that *“participation in regional and international harmonisation programmes of different communities and development bodies, e.g., the EAC-MRH, WHO-PQ, WHO-CRP, Swissmedic etc.”* (P21) enabled the domestication of the model law. This point is supported by another participant (P7) who stated that their NRA’s participation in the EAC medicines regulatory harmonisation initiative as per treaty and protocols of the establishment of the EAC was a facilitator of the domestication process.

#### 4.3.2. External policy and incentives

The external policy and incentives construct is a broad construct that includes external strategies to spread interventions, including policy and regulations (governmental or other central entity), external mandates, recommendations and guidelines, pay-for-performance, collaboratives, and public or benchmark reporting. In several countries, the legislation for medicines regulation was updated due to the desire to align with the AU Model Law and provide for regulatory functions that were missing in the legislation. In addition to wanting to align with the AU Model Law, one country reported to be amending their legislation to close gaps that were identified when their regulatory system was assessed using the WHO Global Benchmarking Tool (P14). Another country feels that they have an *“obligation to align with international recommendations”* (P22) and are therefore updating their legislation.

### 4.4. Inner setting

The study findings are consistent with two out of five inner setting constructs.

#### 4.4.1. Implementation climate

Implementation climate is the absorptive capacity for change, shared receptivity of involved individuals to an intervention, and the extent to which use of that intervention will be rewarded, supported, and expected within their organisation. Implementation climate has the following six sub-constructs: tension for change, compatibility, relative priority, organisational incentives and rewards, goals and feedback, and learning climate. The study findings are consistent with one out of the six sub-constructs.

##### 4.4.1.1. Tension for change

Tension for change refers to the degree to which stakeholders perceive the current situation as intolerable or needing change.

###### 4.4.1.1.1. The establishment of a new regulatory authority

One of the reasons why AU Member States were updating their legislation for medicines regulation is due to a desire to establish a new regulatory authority or restructure the existing one as the current authority (or the absence of one) was deemed to require changing. This point is supported by a Francophone participant who stated that the motivation to update the legislation for medicines regulation in their country was due to the need for “*an autonomous and independent medicines regulatory authority for greater consumer protection against counterfeit, spurious or falsified pharmaceutical products and the illicit market”* (P23). Another respondent (P18) stated that the reason for updating the existing legislation in their country was political for their government which wanted to strengthen legislation and regulation of the pharmaceutical sector, as well as create an NRA.

In African countries where a regulatory authority already exists, legislation was updated to transform the existing institution. For instance, in Ethiopia, the previous regulations were for all health products, professionals, and services and all these were regulated by one authority, the Food, Medicine and Healthcare Administration and Control Authority (FMHACA). When the legislation was updated, it resulted in the Ethiopian Food and Drug Administration (EFDA), a regulatory authority with a mandate to regulate food and drugs. A similar situation occurred in Zimbabwe where the legislation was updated to change from the Drugs Control Council (DCC) and the Zimbabwe Regional Drug Control Laboratory (ZRDCL) to the Medicines Control Authority of Zimbabwe (MCAZ) in 1997. In Ghana, the legislation was updated to provide for a more comprehensive law on public health, make the existing legislation more responsive to contemporary health issues and to upgrade the then Food and Drugs Board to a Food and Drugs Authority, and in Tanzania, there was a desire to shift the regulation of food and cosmetics to the Tanzania Bureau of Standards, and the Tanzania Food and Drugs Authority (TFDA) became the Tanzania Medicines and Medical Devices Authority (TMDA).

###### 4.4.1.1.2. Support for regulatory harmonisation and international collaboration

The desire to have legal provisions at the national level that allow regional harmonisation and international collaboration is one of the enabling factors that featured prominently in this study. As one participant said, domesticating the model law is *“above all a question of the desire to have legal provisions which make it possible to protect public health through, in particular, regional harmonisation*, *and international collaboration”* (P20) and another spoke of the *“need for harmonisation of pharmaceutical regulations”* (P23).

###### 4.4.1.1.3. The desire to have an efficient and effective regulatory system

A participant stated that *“the desire to have an all-encompassing legislation for regulation of health products and technologies”* (P12) and *“the policy direction to set up a single regulatory authority for regulation of all health products and technologies”* (P12) were enabling factors for the domestication and implementation of the model law. This was supported by another participant that stated that model law adoption was enabled by *“the desire to strengthen legislation on medicines and health products on the African continent”* (P22). Additionally, in one country *“the regulatory framework is constantly being reinforced which has helped to domesticate the model law”* and in another, there is a *“breakthrough movement towards the achievement of (WHO) maturity level 3”* (P6). All these points illustrate the tension for change that must exist for implementation of interventions. Timing also enables model law domestication as one participant noted that in their country, the AU Model Law came at a time when their NRA was ready for amendments (P10). Furthermore, the presence of gaps in the current Act (P17) and the desire to have an appropriate law including all the regulatory functions of an NRA facilitated the adoption of the model law.

#### 4.4.2. Readiness for implementation

Readiness for implementation refers to tangible and immediate indicators of organisational commitment to its decision to implement an intervention. There are three sub-constructs under this construct, and these are leadership engagement, available resources, and access to knowledge and information. The study findings are consistent with two of the three sub-constructs.

##### 4.4.2.1. Leadership engagement

Leadership engagement refers to the commitment, involvement, and accountability of leaders and managers with the implementation. Political will and leadership are considered by the participants to be enabling factors for the domestication and implementation of the AU Model Law. For instance, one participant reported that in her country, they had *“political support from our parent Ministry and the Government”* (P8). Another participant attributed successful domestication of the model law to *“goodwill from the (NRA) management”* (P10) and *“the leadership of the CEO”* (P10).

##### 4.4.2.2. Available resources

Available resources refer to the level of resources dedicated for implementation and on-going operations, including money, training, education, physical space, and time.

The process of domesticating and implementing a law requires resources and participants stated that the availability of both financial and human resources enabled the process in their respective countries.

### 4.5. Process

The constructs in this domain are planning, engaging, executing, and reflecting and evaluating. The study findings are consistent with two out of four process constructs.

#### 4.5.1. Engaging

Engaging means attracting and involving appropriate individuals in the implementation and use of the intervention through a combined strategy of social marketing, education, role modelling, training, and other similar activities. There are four sub-constructs under engaging, and these are opinion leaders, formally appointed internal implementation leaders, champions, and external change agents. This research study found that one of the enabling factors for the domestication and implementation of the AU Model Law is the presence of advocates, facilitators, or champions for the cause. These can be either internal actors (i.e., NRA staff) or external actors (i.e., persons who are not NRA staff).

In terms of internal facilitators, champions, or advocates, 76% of the NRAs (*n* = 16) reported that they had internal facilitators in the process of AU Model Law domestication. Most of the internal facilitators were from the legal department/team, and they performed various roles. In one NRA, the legal member of the NRA facilitated the drafting of the layman draft and in another, the legal department facilitated the process and communicated with responsible government offices. In addition, the NRA’s lawyer of one AU Member State is said to have worked with the Head of the NRA and through explanatory memoranda and meetings, they brought to the attention of the Authority the importance of domesticating such a law. In another country, the legal team worked with the technical departments and identified implementation challenges and ensured that the process addresses them. Lastly, the legal unit of one NRA captured all the new additions and ensured that the Medicines and Allied Substances Bill was submitted for review.

The second most identified internal facilitators were technical staff of the NRA who are said to have been instrumental in the drafting of the laws. For instance, one respondent highlighted that the NRA technical staff played a role in the *“development of the amendment Bill and submission of comments in support of the Bill during the public participation stage of the Bill”* (P12). Technical staff also organised and participated in meetings as well as advocated for the domestication of the law to the Minister of Health, Cabinet and Parliament.

The Head of the NRA as well as the Governing Board were also advocates for the domestication and implementation of the AU Model Law. One Head of Agency reported in the survey that they *“participated in member state committee and stakeholders’ meetings as a representative of the NRA and Ministry of Health (MOH)”* and another stated that they *“developed the draft law, presented in Board meetings, Ministry of Health, stakeholders’ meeting, inter-ministerial Permanent Secretaries committee, AG (Attorney General) chamber, Cabinet of Ministers, and House of Representatives”* (P7). In one African country, the Governing Board, and the Head of the NRA *“championed the domestication of the law through organising consultative stakeholder workshops and working groups to ensure that (the country’s) Regulatory Health laws can effectively respond to contemporary health issues”* (P8). Other participants stated that their Heads of Agencies played a crucial advocacy role at the level of the Ministry of Health and *“monitored the drafting of the model law in accordance with the health code.”*

The focal person for the regional medicines regulatory harmonisation initiative and the Public Health Commissioner of the country were also identified as internal facilitators of the process. In sum, *“having people that understand the importance of including the missing provisions in the national legislation”* (P26) is an important enabling factor for the domestication and implementation of the AU Model Law.

In terms of external facilitators, two thirds (*n* = 14) of the NRAs had external facilitators, advocates, or champions involved in the AU Model Law domestication and implementation process. The Ministry of Health was the most mentioned external facilitator in this process and its role differed from one country to the next. In some cases, it played the crucial role of *“communicating with the Attorney General and other government offices”* (P6) and submitting *“the bill to the office of the Attorney General and thereafter presenting their input during public participation.”* After the Ministry of Health, the most mentioned external facilitator was the AUDA-NEPAD which worked with RECs to raise awareness and engage political and senior leadership on the AU Model Law. The AUDA-NEPAD is also reported to have trained *“the actors involved in pharmaceutical regulation on this law and its implementation”* (P25).

Some countries had more external facilitators than others. One Anglophone and one Francophone country in particular stand out as they had support from several external institutions. The former listed the Ministry of Health, the AUDA-NEPAD and the WHO African Regional Office as external facilitators in their domestication process, and these actors *“provided technical and financial support in ensuring that (the country’s) legislation on medicines aligns with the AU Model Law”* (P8). In the Francophone country, the Ministry of Public Health and the Fight against AIDS, the Ministry in charge of East African Community Affairs, the AUDA-NEPAD, the East African Community (EAC), the World Bank, and the WHO country office were all external facilitators in the domestication and implementation process. Their roles were *“advocacy for the establishment of a Pharmaceutical Law based on the AU Model Law, financing meetings, and sensitising different institutions in the country”* (P21). One participant (P23) mentioned that in their country, the West African Economic and Monetary Union (WAEMU), the Agence Française de Développement (AFD), and WHO were external facilitators, and their roles were providing technical and financial support for the adoption of the AU Model Law. Other less common external facilitators mentioned by study participants are the RECs (namely SADC and the EAC), WHO country offices, non-governmental organisations (P13), the local pharmaceutical industry (P10), and academia (P10).

#### 4.5.2. Executing

Executing is carrying out or accomplishing the implementation according to plan.

##### 4.5.2.1. The process of AU Model Law domestication and implementation

The process of domesticating the AU Model Law differs from one country to the next.

Most respondents indicated that in their country, the process of domesticating the model law begins with the NRA’s legal unit and the legal committee reviewing the existing legislation against the AU Model Law. Afterwards, the NRA’s legal unit and legal experts develop a draft law which is then reviewed by the Legal Committee. The draft law is then circulated to stakeholders for comments and final revisions are made by the NRA’s legal unit to incorporate any comments. The Legal Committee has the responsibility to approve the final draft law and it is then submitted to the Minister of Health for approval. Next, the draft law goes to the Attorney General’s office for approval, and then to Cabinet, and finally to Parliament. If Parliament approves of the draft law, it is then published in the government gazette.

One participant stated a process that has less steps compared to other countries. For them, *“the Authority prepares a draft and then it is circulated to stakeholders who provide comments. Afterwards, the Board of the NRA reviews a draft that has incorporated stakeholders’ comments, and the Legal Committee then draft penalties, and the draft law goes to Parliament for approval”* (P2).

In one country, they circulate the Bill to stakeholders, both locally and regionally. This is done after the existing legislation is reviewed against the model law by the NRA’s Legal Committee and stakeholders from industry and professional groups and drafting instructions have been submitted to the Attorney General for drafting of the amendment Bill. Once the local and regional stakeholders have reviewed the draft law and provided their comments, it is submitted back to the Attorney General for final draft. This will be approved by Cabinet and then subjected to public comment. From there, it will be approved by Parliament and published in the gazette with effective commencement date (P15).

In another country that has domesticated the model law, the NRA’s staff and the legal unit of the Ministry of Health reviewed the existing legislation against the model law and then they drafted a Bill with the support of various partners. A high-level meeting was then organised by the East African Community in the presence of other partners (i.e., the AUDA-NEPAD, WHO, and the World Bank) to advocate for the domestication of the AU Model Law. Next, the draft law was finalised by the legal team of the Ministry of Health and reviewed and approved by the National Service of Legislation (SNL). The draft law was then circulated to stakeholders for comments, after which final revisions were made to incorporate the comments. The SNL then approved the final draft, and the Bill was returned to the legal unit of the Ministry of Health. The draft law was then approved by the Minister of Health followed by the Council of Ministers. The Minister of Parliament then visited EAC countries that have set up NRAs. Next, Parliament approved the draft law, and the law was promulgated by the President of the Republic. The last step was publication of the new law in the official gazette (P21).

##### 4.5.2.2. Challenges encountered in AU Model Law domestication and implementation

The challenges or barriers encountered in the process of domesticating and implementing the AU Model Law include the lack of human and financial resources, competing priorities at the national level, overlapping roles of government institutions, and the process of amending/repealing laws being slow and lengthy.

###### 4.5.2.2.1. The lack of human and financial resources

A total of 27% (*n* = 7) of participants stated that one of the challenges they encounter in adopting the model law is the lack of competent human resources. There is *“insufficient human resources in quality and quantity”* (P21), and in one country, there is *“inadequate funding and lack of competent human resources, especially pharmacists”* (P13).

As the model law can result in the establishment of a regulatory authority and the widening of the scope of regulatory functions, participants also stated that domestication of the model law causes *“resource constraints”* (P10) as *“more resources in terms of office space and human resources”* (P10) are needed. Another participant stated that *“the functionality of the pharmaceutical regulatory agency once created can be a major challenge due to the lack of human resources in quantity and quality, and of the infrastructure to house the headquarters of the agency”* (P20).

###### 4.5.2.2.2. Competing priorities at the national level

In some countries, there are competing priorities at the national level which impede the domestication and implementation of the model law. One participant stated that in their country, there were *“many concurrent legal reforms to align Acts with the new Constitution and Medicines and Allied Substances Control Bill did not make it top priority”* (P16).

Other challenges include the lack of *“political will and acceptance by the public”* (P2), *“lack of political will and resources to support legal reform”* (P11) as well as *“lack of prioritisation and availing of financial resources”* (P26).

###### 4.5.2.2.3. Overlapping roles of government institutions

One participant (P7) reported that in their country, there is an overlap in legislation for the NRA, Bureau of Standards, Chief Government Chemist, and for agriculture and livestock. Therefore, when the time came to adopt the model law, there were differing views regarding the AU Model Law components that should be domesticated and the types of products that the NRA should regulate. In another country, a similar challenge emerged as there are *“overlapping missions in different texts”* (P21).

###### 4.5.2.2.4. The process of amending/repealing laws is slow and lengthy

A participant explained that *“a key challenge is that the steps involved from drafting of the amendments to endorsement of the updated legislation involve different stakeholders. The urgency of moving forward with the process differs from stakeholder to stakeholder thus the process may not be as fast as may be desired by for instance the NRA”* (P26). In one country, they stated that there is now *“blockage of the process at the level of the Ministry of Health”* (P19) and in another there is “*misunderstanding of different ministries and government institutions”* (P21).

It is also difficult to have *“full engagement of stakeholders in a timely manner”* (P15) and as a result, *“consultations had to be extended several times to ensure inclusivity”* (P15).

##### 4.5.2.3. Solutions to overcome the challenges encountered in domesticating and implementing the model law

To address the challenges encountered in AU Model Law domestication and implementation, NRAs advocated for their governments and various stakeholders to adopt the model law, and they had frequent communication, consultations, and discussions on the importance of domestication of the AU Model Law. One participant stated that *“political will and resources are key and usually inadequate so more advocacy to governments especially Ministries of Health and Justice for the full domestication would greatly help”* and another said that they held *“stakeholders’ consultations of political leaders including parliamentarians and Ministers of state on the importance of implementing the AU Model Law”* (P11). In addition, NRAs requested assistance from development partners such as the World Bank, through the AUDA-NEPAD, the AU, WHO, RECs, and other international bodies. Furthermore, in countries where the AU Model Law’s domestication was challenging due to overlaps in roles, duties, and responsibilities of the NRA and another government institution, a solution that was being considered was *“the demarcation of roles, duties, and responsibilities”* (P7). In one country, the NRA organised *“courtesy visits to exchange with the institutions concerned in order to understand the roles, responsibilities and limits of each”* (P21). NRAs also sought funds to support the process (P17) and in cases where the process was slow and lengthy, timelines were extended to allow industry and stakeholders time to provide input (P15). One country with human resource challenges is advocating for the government to scale up the number of students who study pharmacy as well as to recruit more pharmacists (P13).

## 5. Discussion

All the countries in this study have an NRA or an administrative unit that is responsible for the regulation of medical products and nearly all the NRAs that participated in this survey stated that there is legislation in place for medicines regulation. In some countries, legislation for medicines regulation dates back as far as 1957 whereas in other countries, legislation first came into effect as recently as 2020. Most countries have also updated their legislation at least once and some are currently doing so. These findings are consistent with those reported by Ndomondo-Sigonda et al. (2). Additionally, this study found that countries update their legislation for medicines regulation for reasons such as the desire to establish a new regulatory authority, to transform the existing regulatory authority, or to align their legislation with the AU Model Law and international best practices. It is worth noting that a third of the NRAs that participated in this study reported that they have domesticated the model law and over 90% are yet to do so despite the AMRH initiative, within the framework of the AU Pharmaceutical Manufacturing Plan for Africa, having set a target in its AMRH Strategic Framework (2016–2020) to domesticate the AU Model Law in at least 25 AU Member States by 2020. This target has not been achieved. Our study found that in order to achieve this target, there must be support for regulatory harmonisation and international collaboration in AU Member States as well as the availability of resources, the presence of political will and leadership, the desire to have an efficient and effective regulatory system, and the presence of facilitators/champions for the cause.

As previously reported, the pharmaceutical sector is incredibly dynamic, characterised by several distinct stakeholders with diverse interests, and this creates a scenario where pharmaceutical policy cannot have a “one size fits all” approach (19). The process of policy development is almost exclusively a national matter and will differ among countries and regions with disparate levels of income (18). Countries and RECs with similar objectives may need different policies, taking into consideration their respective starting positions, pre-existing laws and regulations, and implementation capacity (19). Therefore, it is not surprising that our study countries had different processes of AU Model Law domestication and they either domesticated the model law in full or they are conducting a partial domestication. It is important to have all stakeholders involved early in the process as it will result in a stable system that can guarantee access to and rational use of medicines (18). Countries are also forced to develop a transparent framework during the policy development process so that stakeholders understand their roles and responsibilities (18). Additionally, African countries with the greatest disease burden also have the most resource limited NRAs (20). NRA regulators in 26 African countries were interviewed by WHO assessment teams which found that across the board, there exists weak management structures and processes, a severe lack of qualified personnel, and scarce resources (20). Therefore, it is at this early stage when all stakeholders are involved that national priorities need to be defined based on a balance between meeting the needs of patients and ensuring the effective use of available resources (18). Resources needed for policy revisions should also be allocated at the beginning of the policy development process (18).

Policy implementation is a major problem in low-income countries (21). Failures in achieving the desired policy goals can be attributed to inadequate resources, a lack of communication bridging research to policy, an absence of a strategy, governance instability and a lack of political commitment (21). In our study, the challenges or barriers encountered in the process of domesticating and implementing the AU Model Law include the lack of human and financial resources, competing priorities at the national level, overlapping roles of government institutions, and the process of amending/repealing laws being slow and lengthy. Hoebert et al. (18) report similar challenges in pharmaceutical policy implementation and contend that the process requires sufficient staff with appropriate technical and professional capabilities (18). They also found that some policies that affect medicines contradict or undermine others and argue that the absence of an integrated national policy is unsatisfactory from a public health standpoint (18). However, they do admit that the process of deciding which functions fall into which area is a complex one, and the decision to proceed as well as the subsequent success of implementation is dependent on political support and capacity at the local level (18). Furthermore, shortcomings in regulatory performance, lack of access to medical products, and irrational use of medicines may exist despite the existence of a comprehensive policy document (18).

In our study, one of the ways that participants addressed the challenges that they encountered in AU Model Law domestication and implementation was to approach their governments and various stakeholders and lobby them to adopt the model law, and they had frequent communication, consultations, and discussions on the importance of domestication of the model law. Additionally, NRAs requested assistance from development partners and other international bodies. These partners have prior involvement in the regulatory landscape in Africa, particularly in the AMRH initiative. Literature confirms that the involvement of external stakeholders and garnering high-level political support enables the development and implementation of a policy. For instance, when South Africa was developing its first single National Medicines Policy, it invited the WHO to participate from the start and this high-level political support resulted in the final policy document in 1996 (18). The support also ensured the successful implementation of most of the national components of the policy in the years that followed (18).

In this study, some of the perceived benefits of domesticating and implementing the model law are to enable cooperation with other NRAs, to harmonise regulatory systems and to facilitate mutual recognition between and amongst countries. This finding can be explained by Ahonkhai et al.’s (20) analysis which found several complexities in the current regulatory system such as disparate NRA standards and requirements in low- and middle-income countries (LMICs). This leads to additional work and duplicative efforts for manufacturers when submitting marketing authorisation applications in different African countries (20). Taking into consideration the limited commercial returns in LMICs, eliminating duplicative efforts and adopting a common set of technical product registration requirements makes sense (20). The finances and time needed to write, re-write, and manage applications from one country to another remains a disincentive for manufacturers (20). In Europe, concerns about greater consistency and optimised access to quality-assured medicines was one of the strongest motivators for developing a unified pharmaceutical regulation approach that exists today in the European Union (20). Additionally, it is perceived by NRAs that model law domestication will result in an increased number of registered medical products. According to literature, pharmaceutical manufacturers tend to spread submission of new products to African NRAs over several years and Ahonkhai et al. (20) identified a number of potential root causes of this situation. These root causes include the fact that multi-national companies did not typically prioritise early registration and introduction of innovative medical products into low-income countries due to limited commercial potential in these markets (20). Secondly, low-income countries have varying requirements and legislative frameworks that limit manufacturers’ ability to submit a single dossier concurrently to these countries (20). The spread is further exacerbated by the enormous resources required to prepare unique submissions for each country as well as respond to the queries from each individual NRA (20). Therefore, some countries experience long waits before they receive marketing authorisation applications (20). It is evident that NRAs hope that the model law will address the *status quo*.

An interesting perception stated by a participant in our study was that domesticating and implementing the model law would enable the regulated community to clearly understand their roles. This is important as we note that in Sri Lanka, the first two attempts (in 1991 and 1996) to develop a National Medicines Policy failed due to strong lobbying against it by the private pharmaceutical industry even though they had participated as a stakeholder (18). This demonstrates how it is important to get buy-in from the pharmaceutical industry and have them clearly understand the importance of pharmaceutical policies and their roles. It was also interesting to see a participant in our study making the link between AU Model Law domestication and enabling them to participate in the realisation of the AMA project. This is in line with literature that states that the long term goal of the AMRH initiative is to establish the AMA, which will have the mandate of overseeing the registration of specific medical products and coordinating regional harmonisation systems in Africa (5, 22). Therefore, the development of the AU Model Law is interpreted within the context of these overarching efforts towards regulatory harmonisation in Africa (5). These efforts in regulatory systems harmonisation are a pivotal aspect when laying the foundation for establishing a single continental regulator (5, 22–26).

In this study, most respondents stated that there were no perceived disadvantages of domesticating the model law. However, those that did considered the lengthy process of amending existing Acts to be a disadvantage. As previously reported, law amendments are a lengthy process that requires two vital steps: (i) ensuring precise technical wording of the policy, and that it is consistent with other national laws and can be implemented; and (ii) passing the policy amendments through the formal, established, legally required administrative processes (11). In principle, solutions to address the challenges related to pharmaceutical policy and regulatory reform are relatively straightforward; however, the implementation aspect of the process is very much complicated (27). Another perceived disadvantage uncovered by this study is that since the provisions of the AU Model Law will be applied across the region, there is the possibility of countries with robust regulatory systems relying on data from other AU Member States by reason of harmonisation and this data might not be up to standard. This is a valid concern as only 7% of African NRAs have moderately developed capacity to undertake medicine regulatory functions and over 90% have minimal-to-no capacity (28). African NRAs are also reported to lack competent regulatory professionals, have high staff turnover, inadequate staffing numbers relative to the high workload, low diversity of scientific expertise, perennial backlogs, limited financial resources, poor regulatory infrastructure and they encounter challenges when they try to collaborate with other NRAs in the region (28). Additionally, only five African countries have NRAs that operate at WHO Maturity Level 3. These countries are Tanzania, Ghana, Egypt, Nigeria, and South Africa. Tanzania, Ghana, and Nigeria have maturity level 3 status for medicines and imported vaccines, Egypt’s maturity level 3 status is for vaccines regulation (locally produced and imported), and South Africa’s is for vaccines (producing) (29, 30). The NRAs of these five countries represent effective regulatory systems on the African continent. Other African NRAs are currently being assessed (29, 30).

Another perceived disadvantage of domesticating the model law stated in this study is that the expanded mandate brought about by the AU Model Law may not be affordable. As it stands, many LMICs cannot finance their public health needs and their NRAs are particularly vulnerable (10). African NRAs have annual budgets that are relatively small and a large portion of the budget is reserved for operational costs. This leaves an even smaller amount for infrastructure development and salaries (31). Studies conducted by Ndomondo-Sigonda et al. (31) and Sithole et al. (32) in the EAC region and SADC, respectively report that African NRAs use different financing models. Funds are generally obtained from their governments, fees for services provided (such as fees for registration, annual product maintenance, plant audits, licensing of premises, and import permits) and/or from donors (2, 10, 31, 32). In some African countries where the NRAs rely on funding from government, all fees are paid directly to Treasury. These fees are not redistributed and the funds allocated to NRAs by the respective governments are not transferred on time (31). While most African NRAs levy fees, the fees are usually arbitrary amounts that are not commensurate with their regulatory workload or value-added activities (10). This results in market entry barriers, and it hinders post-marketing quality surveillance, impedes reliance efforts, and prevents potential financial sustainability (10). Based on these factors, NRAs can neither pay competitive salaries nor sustainably finance workforce capacity development activities. For there to be effective and long-term functioning of NRAs, goals therefore need to be clearly defined and sustainability, in terms of human and financial resources, must be institutionalised. There is also a need for NRAs to be granted financial autonomy through clear government policies and legal frameworks that allow them to collect and use fees for services rendered. Furthermore, a fee structure that is commensurate with regulatory workload must be developed by NRAs. Fortunately, African countries can domesticate the AU Model Law which will assist them to amend, repeal and/or enact laws that grant NRAs the power to levy, collect and use fees for the services that they offer (10). All this should improve the financial stability, functional efficiency, and accountability of NRAs. Lastly, in this study, all participants who stated that they have implemented the AU Model Law reported that there have been no disadvantages to its implementation. It is important that mechanisms for implementation and monitoring are created after the official adoption of a policy (18).

Based on the findings of this research study, the following recommendations are made:

1. Governments should fast-track the process of amending existing Acts to incorporate key components of the AU Model Law. They should also provide technical and financial support to their NRAs and African medicines regulatory harmonisation initiatives.
2. Governments should demarcate roles, duties and responsibilities of institutions in order to avoid any overlaps and any legislation/regulations that contradict each other must be amended or repealed.
3. African NRAs must participate in regional and international harmonisation programmes of different communities and development bodies (e.g., the WHO Prequalification Programme, the WHO Collaborative Registration Procedure, and the Swissmedic Marketing Authorisation for Global Health Products procedure) as this is an enabler for AU Model Law domestication and implementation. It also provides an opportunity for African regulators to improve their regulatory expertise and capacity.
4. The formalisation of twinned review and exchange programmes to enable African regulators to work alongside reviewers from SRAs is recommended. We further recommend that African regulators be involved in the process of developing regulatory guidelines as this is an unexplored avenue to build capacity and expertise.
5. NRAs cannot pay competitive salaries or sustainably finance workforce capacity development activities. Therefore, for effective and long-term functioning of NRAs, goals need to be clearly defined and sustainability, in terms of human and financial resources, must be institutionalised. NRAs also need to be granted financial autonomy through clear government policies and legal frameworks that allow them to collect and use fees for the services they offer. Furthermore, NRAs must develop a fee structure that is commensurate with their regulatory workload. This can all be achieved by domesticating and implementing the AU Model Law which will assist AU Member States to amend, repeal and/or enact laws that grant NRAs the power to levy, collect and use fees for services that they offer.

### 5.1. Study limitations

It is possible that the experiences of the countries that were excluded from the study are different from the included study participants. The use of in-depth interviews as a data collection method may have revealed more themes that are consistent with the Consolidated Framework for Implementation Research.

## 6. Conclusion

Officially endorsed in January 2016 by AU Heads of State and Government, the AU Model Law on Medical Products Regulation aims to harmonise regulatory systems, increase collaboration across countries, and provide a conducive regulatory environment for medical product/health technology development and scale-up. However, not all African countries have domesticated the non-prescriptive model law. This research found that the perceived benefits of model law implementation include enabling the establishment of an NRA, improving NRA governance and decision-making autonomy, strengthening the institutional framework, having streamlined activities which attract support from donors, as well as enabling harmonisation, reliance, and mutual recognition mechanisms. The factors enabling domestication and implementation are the presence of political will, leadership, and advocates, facilitators, or champions for the cause. Additionally, participation in regulatory harmonisation initiatives and the desire to have legal provisions at the national level that allow for regional harmonisation and international collaboration are enabling factors. The challenges encountered in the process of domesticating and implementing the model law are the lack of human and financial resources, competing priorities at the national level, overlapping roles of government institutions, and the process of amending/repealing laws being slow and lengthy. Addressing these challenges will result in a harmonised legal environment for medicines regulation in Africa, improve the functioning of African NRAs, and be an important enabler for the effective operation of the African Medicines Agency, the establishment of which will be another crucial step towards regulatory harmonisation on the continent.

## Data availability statement

The raw data supporting the conclusions of this article will be made available by the authors, without undue reservation.

## Author contributions

BN designed the study, collected and analysed the data, and wrote the first draft of the manuscript. AD and KW designed the study, interpreted the results, and reviewed subsequent drafts of the manuscript. All authors contributed to the article and approved the submitted version.
